# Preparing for Takeoff: Addressing In-Flight Challenges Faced by Persons Living with Dementia and Travel Companions

**DOI:** 10.1093/geroni/igaf122.2485

**Published:** 2025-12-31

**Authors:** Colleen Peterson, Renee St. Louis, Tamara Statz, Maria O’Reilly

**Affiliations:** University of Michigan Transportation Research Institute, Ann Arbor, Michigan, United States; University of Michigan Transportation Research Institute, Ann Arbor, Michigan, United States; Vibrant Living, St. Paul, Minnesota, United States; CQUniversity Australia, Bundaberg, Queensland, Australia

## Abstract

Addressing the challenges faced by travelers with dementia and their companions requires developing accessible services based on inclusive principles, dementia awareness, and a willingness to listen to the voices of those living with dementia. Although increasing awareness of the needs of travelers with dementia has seen positive changes within airports globally (e.g., the sunflower lanyard), the needs and experiences of travelers with dementia in-flight are less well understood. This poster combines findings from two similar studies conducted in the USA and Australia exploring in-flight issues from the perspective of travelers with dementia (n = 55), their companions (n = 217). We share the consistent themes on in-flight challenges that were found in both studies, with additional insight from flight crew in the Australian study (n = 21). In-flight challenges included anxiety, confusion, toilet access and use, awareness of available assistance, negative flight crew interactions, and seating arrangements. We share participants’ own strategies for preventing or mitigating the issues they have faced and make additional recommendations for air travel guidelines to support safe travel.

